# Anything goes for participant, patient and public involvement in youth mental health research

**DOI:** 10.1002/jcv2.12258

**Published:** 2024-07-03

**Authors:** Marian J. Bakermans‐Kranenburg, Marinus H. van IJzendoorn

**Affiliations:** ^1^ ISPA – University Institute of Psychological Social and Life Sciences Lisbon Portugal; ^2^ Research Department of Clinical, Educational and Health Psychology UCL London UK; ^3^ Facultad de Psicologia y Humanidades Universidad San Sebastián Concepción Chile

**Keywords:** cooperative practitioners‐researchers model, participatory action research, PPI, replicability, translation, transparency

## Abstract

**Background:**

Participant and Public Involvement in youth mental health research aims at making research more responsive to the needs of youth struggling with mental health issues, their parents, and mental health professionals and other stakeholders. Do characteristics of Patient and Public Involvement (PPI) in youth mental health research align with transparency and replication prerequisites as necessary conditions for translation? Relatedly, the question is addressed whether co‐authorship should be assigned to youth involved in the study.

**Methods:**

Here we address these questions re‐visiting 50 PPI studies included in two recent systematic reviews of PPI on characteristics that are pertinent to questions about transparency, replicability, translatability, and co‐authorship in PPI research.

**Results:**

Almost two‐third of the studies on youth mental health incorporating PPI translate their results to policy or practice, mostly as recommendations but sometimes also by dissemination of (online) interventions. At the same time the authors of a substantial majority of the studies (70%) also suggest the need for further work on their results, for example, in randomized controlled trials to validate the outcome of their exploratory inquiry. Only a quarter of the studies using PPI met the conditions for replicability, thus a majority of the PPI studies suggest premature translation of results. Authorship to involved participants was assigned in 24% of the studies.

**Conclusions:**

“Anything goes” for PPI in an exploratory stage to generate fruitful hypotheses. Translation of the findings of PPI studies however require a firm evidence base of replicated results. Radical merging of research and action in participatory action research seems incompatible with replicable and therefore translatable inquiry. Assigning co‐authorship to PPI representatives is often at odds with current guidelines for authorship. More evidence from randomized trials on the translational impact of PPI is needed before grant foundations should require PPI in grant proposals.

## Graphical abstract

In the paper we argue that “Anything goes” (Feyerabend) when using PPI in an exploratory way to generate fruitful hypotheses. Translation of the findings of PPI studies however require a firm evidence base of replicated results. Radical merging of research and action in participatory action research seems incompatible with replicable and therefore translatable inquiry. Assigning co‐authorship to PPI representatives is often at odds with current guidelines for authorship. More evidence from randomized trials on translational impact of PPI is needed before grant foundations should expect or even require PPI in grant proposals.

**Key points**

- Patient and Public Involvement (PPI) should in a Wittgensteinian way be defined as a continuum from participants being passive data suppliers to playing a blended role of activists and co‐researchers.
- Whether PPI facilitates effective and responsible translation of research to policy or practice has not yet been experimentally demonstrated.
- Re‐coding of 50 PPI studies from two recent systematic reviews showed that the majority lacked transparency of the PPI component and jumped from non‐replicable results to non‐responsible translation.
- Anything goes for participants in the exploratory context of discovery who may even lead the way, but in the context of justification trained researchers should have the final say.
- PPI representatives cannot be named as co‐authors according to current scientific authorship definitions and guidelines.

## INTRODUCTION

In biomedical and mental health research, “Patient and Public Involvement” (PPI) or “Participant, Patient, Practitioner, or Public Involvement” (P4I), is becoming more and more important as a way to make research more responsive to the needs and priorities of research targets and stakeholders. PPI would improve the quality and relevance of research by ensuring that the research question is relevant and leads to actionable answers. Participants, patients, practitioners and other representatives of the public are invited to be included in designing and conducting research and translating outcomes to policy or (clinical) practice. Overall, the sympathetic aim is to make science and its results more accessible to the individuals whose lives and interests are addressed. But should the voice of the participants and other stakeholders be heard in all stages of the research cycle? Our answer is no: Their voices might be crucial in the exploratory context of discovery of fruitful hypotheses but should not be included in the context of justification, that is, in the stringent replicable tests of bold conjectures.

### PPI as co‐researchers

Historical roots of PPI may be traced back to participatory action research in the sixties and seventies of the last century, inspired by the German movement toward the critical theory and methodology of “Handlungsforschung” (literally translated as “action research”, see Van IJzendoorn & Van der Veer, 1983). The idea was that researchers should join forces with the public, for example, factory workers or school teachers, to facilitate social change by a cyclical intertwining of research and action. Researchers should not have a privileged position as producers and gatekeepers of knowledge but share their expertise and responsibilities from the very start of designing a study all the way to the implementation of change in the larger community, and back. This was reflected in the ideas of Hart (1992) who argued that research and action should blend into action research, and that individuals should be both researchers and participatory activists.

**FIGURE 1:**
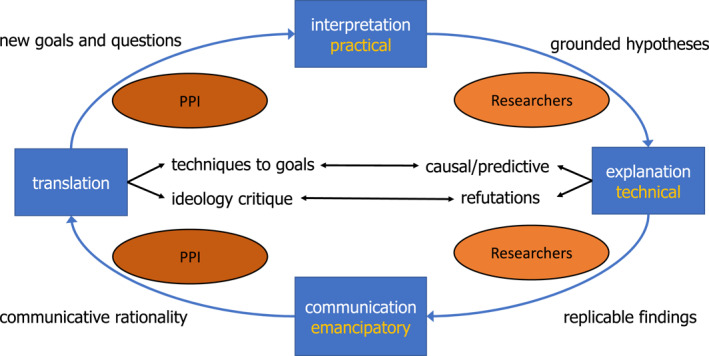
Cooperative practitioners‐researchers model.

One of the historical roots of PPI was action research, popular in post‐WOII Europe, and some current variants of PPI as participatory action research still are inspired by this critical movement toward a greater role of research in social change. We consider this type of participatory action research as located at the extreme end of a continuum running from no PPI at all (as is the case in many conventional research projects) via a balanced but clearly differentiated involvement of participants and researchers in specific stages of the research, to the blending of research and activist roles in radical variants of PPI. This blending is for example, advanced by Cornish et al. (2023) in their model of Participatory Action Research as a cyclical process from problem definition to action to observation to reflection back to re‐defining the problem followed by a new start of the action research cycle (see Figure 1, Cornish et al., 2023). Thus, although not all PPI is participatory action research, this research can be visualized at the extreme end of the PPI continuum.

One of the prominent early examples of action research with a high level of youth involvement was the Just Community Project initiated by Harvard developmentalist Lawrence Kohlberg and colleagues (e.g., Power, 1988). Inspired by the theory of moral development (Kohlberg, 1971) one high school in the USA participated in a large‐scale year‐long experiment of promoting the moral climate in the classrooms by installing clear norms of reciprocity, democratic decision making, and discussion of moral dilemmas that were central to Kohlberg's theory of moral development. Close cooperation between researchers, teachers and students served as the basis for the transition of the school to a just community. Despite the absence of a comparison school or comparator group it was concluded that short‐term enhancement of participants' moral reasoning was indeed established. However, any signs of change on the organizational, curricular, or student moral reasoning level had faded away after a year, and no dissemination to other schools was noted (Bakermans‐Kranenburg & Van IJzendoorn, 2019; Power, 1988).

PPI cannot be sharply defined and demarcated from other participatory translational research but may be conceptualized as a Wittgensteinian set of family resemblances, that is “a complicated network of similarities overlapping and criss‐crossing” (Wittgenstein, PI 66, in Biletzki & Matar, 2021). Not the age of the participants is an essential feature of PPI but their active role in various stages of the research process. Participants in PPI go beyond the conventional role of supplier of data, and actively (co‐)shape parts of the research and translation cycle. In the mildest forms of PPI participants only serve in advisory boards for researchers who call the shots. On the other end of the spectrum PPI is prominent in all stages of research, from problem definition to hypothesis testing to interpreting results and translating findings to policy or practice (Ozer, 2016). Participants are co‐researchers and decision‐makers on an equal footing with the researchers (Cornish et al., 2023). But the core assumption shared by all participatory or PPI approaches is the emphasis on participants (youth or adults) as unique experts of their own problem experiences (e.g., Ozer et al., 2020). This expertise would be invaluable for valid research on mental health issues and for initiating effective change for the better.

### Reproducibility and replicability of PPI research

As communicated loudly and clearly in the scientific (Ioannidis, 2005) and non‐scientific media (The Economist, 2013), a replication crisis is raging in the biomedical, biobehavioral and developmental sciences, and it does not skip research focusing on mental health problems in children and adolescents. Numerous research results in (epi‐)genetic, hormonal and neural (pre‐)clinical studies on child mental health could not be replicated in independent research. Some of the causes for lack of replicability are lack of statistical power due to small samples (Elliott et al., 2020; Marek et al., 2022), the abounding use of researcher degrees of freedom and *p*‐hacking as a consequence of the desperate search for significant results (Simonsohn et al., 2014), or the use of ambiguous or oversimplified items for complex psychological constructs in questionnaires such as the Strengths and Difficulties Questionnaire (Runze & Van IJzendoorn, 2023).

The use of various, more or less iconoclast methods and analyses however is no problem in exploratory studies. Following Popper's and Feyerabend's philosophy of science, we argued that in the context of discovery “anything goes” (Feyerabend, 1975; Popper, 1959) as long as the inimitable outcome of this creative process is considered not a scientific finding, but a bold hypothesis and starting point for tenacious efforts to refute the proposed conjecture (Van IJzendoorn & Bakermans‐Kranenburg, 2024). If some promising and potentially translatable result cannot be replicated in a variety of replication efforts, such a result cannot be used in policy or practice because it is in fact a quasi‐result. We cannot trust findings of a pioneering study as if it would be rock solid when it could as well be a false positive finding (perhaps suffering from the so‐called “winner's curse”, see Bakermans‐Kranenburg & Van IJzendoorn, 2014; Errington et al., 2021; Molendijk et al., 2012). A replication crisis implies a translation crisis (Van IJzendoorn & Bakermans‐Kranenburg, 2021).

The National Academies of Sciences, Engineering, and Medicine (2019) defined reproducibility as the possibility to retrace the steps of a researcher using the same dataset, decisions about operationalizations and codes for statistical analyses and find the same results. Replicability goes one step further and concerns the repetition of a study in independent samples with outcomes converging with those of the original study. Replication requires the ability to reproduce what the original researcher did, so reproducibility is a necessary condition for replication, which in turn is a necessary condition for translation to policy or practice (Van IJzendoorn & Bakermans‐Kranenburg, 2024). Some PPI might allow for detailed description and reconstruction in replication studies. But in the more extreme variants of PPI such as participatory action research it might be difficult to document in sufficient detail the steps, decisions and turns taken by a team that accepts diffuse boundaries between researchers and participants, and between data‐collection and application. Another requirement is that the recruitment and characteristics of the sample including the PPI sample is described in sufficient detail. Without reproducibility (also on the level of PPI characteristics, selection, and involvement) no replication.

### PPI in co‐developing interventions

A limited and perhaps traditional way of “giving participants a voice” in mental health research is their involvement in the stages of outlining a question for examination, in co‐developing of the content of an intervention program, or in advising about the best ways to recruit participants or patients (as in O'Farrelly et al., 2021). Such involvement in an early stage of the research cycle does not require reproducibility or replicability because the “bold conjectures” emerging from this stage are meant to be tested in randomized controlled trials (RCTs). For example, in developing our Video‐feedback Intervention to promote Positive Parenting and Sensitive Discipline (VIPP‐SD; Juffer et al., 2017; Van IJzendoorn et al., 2023) parents or professional practitioners were involved in the first stages of the VIPP‐SD development and in the adaptation to specific target groups or settings (immigrant families; parents of twin children; adoptive parents, foster carers, day‐care providers, or teachers). Even though the VIPP‐SD has been proven to be effective across 25 RCTs including more than 2000 families (Van IJzendoorn et al., 2023), we consider PPI involvement in new adaptations of the program necessary.

In developing VIPP‐School (VIPP‐SD for elementary school teachers), teachers made “transformative contributions” (Lloyd et al., 2023) in adapting the observation settings that were used for video feedback, parts of the learning content, and the division of information over the sessions. A qualitative PPI pilot study was instrumental in understanding how teachers and VIPP‐interveners experienced VIPP‐School, and their feedback was used to evaluate the feasibility and acceptability of the intervention program, and to support its further development (Starreveld et al., 2023). Perceptions of teachers and VIPP‐interveners were collected using semi‐structured interviews, logbooks, observations and questionnaires. The PPI study showed that with some adaptations VIPP‐School would be acceptable and deliverable in primary education. Currently, the effectiveness of VIPP‐School is tested in a RCT.

In a similar vein, Christie et al. (2019), developing a digital intervention in ethnic minority groups to learn Cognitive Behavioral Therapy skills via a series of activities and games, organized focus groups and interactive workshops with youth to co‐create an app. The app was developed in consultation with game designers and suggests the user to be on an ocean traveling between islands to learn six evidence‐based skills. The young people's feedback was used to identify and enhance the “look and feel” of the app and maximize its engagement and utility. A prototype of the app was tested by 30 youth using think‐out loud interviews, where they verbalized their thoughts as they moved through the app. Feedback from this process formed the foundation for its further development, for example, it turned out that the prototype users did not appreciate the story line and that they wanted shorter games that could be played more frequently. The authors conclude with the notice that the app and the principles behind its development will be tested in a RCT.

### PPI as co‐authors?

PPI raises an important question about co‐authorship and the roles of PPI representatives in research. The field seems divided in the sense that some papers on PPI studies include the involved youth as co‐authors, whereas other papers acknowledge their contributions in other ways, for example, in a footnote. The International Committee of Medical Journal Editors (ICMJE, 2023) recommends that authorship be based on the following four criteria: (1) Substantial contributions to the conception or design; or the acquisition, analysis, or interpretation of data for the work; AND (2) Drafting the work or revising it; AND (3) Final approval; AND (4) Agreement to be accountable for all aspects of the work. The criteria are meant to include as authors those individuals who made substantive scientific contributions and can be hold accountable. PPI representatives often are “lay” people in terms of their qualifications to evaluate scientific work, whether it is qualitative or quantitative research. The scientific habitus (Lenoir, 2006) scholars acquire during several years of graduate education and long‐term participation in research teams may not be present in most of the (youth) PPI representatives even if they get some further training (Janes, 2016), and it may be too much to hold them accountable for the product. It seems important to discuss the application of the authorship guidelines to PPI studies in order to keep the concept of “author” meaningful and prevent misunderstandings or disappointments for anyone involved in a study.

### The current study

Against this background we address some central questions about the role of PPI in youth mental health research and its translation to policy or practice. Do characteristics of PPI in youth mental health research align with necessary conditions for translation? Are these PPI studies situated in the context of discovery (Popper, 1959) and presented as generating hypotheses for further replication or stringent tests in RCTs? Or do these PPI studies go beyond the exploratory and replication goals and try and translate their results to policy or practice, with or without the involvement of PPI in this stage of implementation? Whether co‐authorship should be assigned to participants or patients who were involved in the study is another complicated issue. Here we address these questions re‐visiting studies included in two recent systematic reviews of PPI (Lloyd et al., 2023; McCabe et al., 2023) on characteristics that are pertinent to our questions about reproducibility, replicability, translatability, co‐research and co‐authorship in PPI research.

## METHODS

### Data‐collection

Two systematic reviews on PPI provided material to be re‐coded with our coding system covering the essential components of translatable studies. In *Health Expectations* (2023) McCabe and colleagues published their systematic review on youth engagement in mental health research. They searched MEDLINE, EMBASE and PsycINFO from the year 2000 to the present with keywords indicating “patient engagement,” “youth” and “mental health” or synonyms. Their goal was to summarize the impacts of youth involvement in the 14 studies that were selected after careful searching and screening this literature. In their *PsyArXiv* (2023) preprint Lloyd and colleagues reported the results of a systematic review of collaborations with children and youth in mental health research. Their goal was to review the ways in which PPI and related approaches (co‐production or co‐design) were commonly incorporated in mental health research involving young participants or patients. EMBASE, MEDLINE and PsycINFO (using the Ovid interface), and Web of Knowledge were searched in May‐June 2023 with key words adolesc* OR child* AND psychiatry OR psychopatholog* OR mental health AND PPI OR co‐produc* OR co‐design*. After careful screening this string yielded 36 relevant studies which were presented in a narrative review. The choice of key words seemed rather similar between the two reviews, but Web of Knowledge was a source only consulted in the Lloyd search. Interestingly, no overlap of studies between the two reviews existed, so we re‐analyzed the total set of 50 PPI studies.

### Coding

In Table 1 the coding system is presented. We rated transparency in terms of explicit description of the participants representing PPI and in terms of their roles in the various stages of the study. If one or both transparency ratings is negative the replicability is necessarily problematic. Translation was divided in two parts, translation to the PPI method, and translation to policy or practice. For translation to the PPI method, we checked whether the authors suggested further tests such as RCTs to fine‐tune or validate the method or to examine how it would work in a different population. Translation to policy or practice was coded when the authors made recommendations for youth mental health care beyond the studied sample and method of inquiry. The ratings were derived from the two systematic reviews as well as from the original papers. For intercoder reliability on replicability and translational aims of PPI studies, the two authors independently coded eight studies (16% of the total set of studies). Intercoder reliability was 83%. Disagreements and tough cases were discussed and consensus reached.

**TABLE 1:** Coding system for rating PPI and translation characteristics.

**PPI as co‐researchers**:
Yes = more than advise: Co‐deciding/producing design, coding, data analysis, and/or interpretation
No = advisory only, no decision making or active involvement in core stages
**PPI as co‐authors**:
Yes = co‐author (check non‐scientific affiliations, notes, etc)
No = no co‐author
**Transparency PPI profile**:
Yes = information on recruitment; sample size; age; gender; SES/Ethnicity
No = missing information on one or more of these 5 descriptors
**Transparency PPI roles**:
Yes = information on involvement in which research stage; what training or supervision
No = no information on either
**Replicability**:
Yes = both transparency ratings for PPI profile and PPI roles are positive
No = one or both transparency ratings are negative
**Translation to further research**:
Yes = advise on PPI method for further testing, e.g., RCTs
No = no advise for further testing
**Translation to policy or practice**:
Yes = advise for practice or policy (broader than PPI method)
No = no advise for policy or practice

Abbreviations: PPI, Patient and Public Involvement; RCT, randomized controlled trial. SES, SocioEconomic Status.

## RESULTS

In Table 2 the results of the ratings are presented with color codings, going from green (potentially adequate) via orange (somewhat disputable) to red (potentially worrisome).

**TABLE 2:** Replicability and translation aims of PPI studies.

No.	First author	Year	Topic	PPI as co‐researchers	PPI co‐authors	Transparency PPI profile	Transparency PPI roles	Replicable	Translation to further research	Translation to policy/practice	Total score
223	Bristow	2020	Playtime	Yes	No	Yes	Yes	Yes	Yes	Yes	5
224	Campbell	2020	Use of emergency department for youth	No	No	No	Yes	No	No	Yes	3
225	Cleverley	2020	Transitioning out of CAMHS	Yes	No	No	Yes	No	No	Yes	2
226	Cleverley	2021	CAMHS clients transitioning to adult services	Yes	No	No	Yes	No	Yes	Yes	3
227	Dewa	2019	Severe mental health illness	Yes	Yes	Yes	Yes	Yes	Yes	Yes	4
228		2021									
229	Ennals	2022	Residential rehabilitation	Yes	Yes	No	Yes	No	No	Yes	1
230	Evans	2017	Synthesis risks inpatients mental health care	No	No	No	Yes	No	Yes	Yes	4
231	Graham	2014	Quality standards youth mental primary care	Yes	No	No	Yes	No	Yes	Yes	3
232	Henderson	2018	Integrated collaborative care model	Yes	No	No	Yes	No	Yes	Yes	3
236	Sheikhan	2021									
233	Kendal	2017	Emotional support needs	Yes	Yes	No	Yes	No	Yes	Yes	2
234	Salami	2021	Mental health care for black youth	Yes	Yes	No	Yes	No	No	Yes	1
235	Samir	2021	Youth advisory research group	No	No	No	Yes	No	Yes	Yes	4
237	Viksveen	2022	Collaboration lead and youth researchers	Yes	Yes	Yes	Yes	Yes	No	No	4
215	Walker	2021	Complex evidence synthesis	Yes	No	No	Yes	No	No	Yes	2
1	Abel	2020	General mental health	No	No	No	Yes	No	Yes	Yes	4
2	Bennett	2022a, 2022b	Trauma and abuse	Yes	Yes	Yes	Yes	Yes	Yes	Yes	4
		2023									
3	Bennett	2021	Help‐seeking	Yes	Yes	No	Yes	No	Yes	Yes	2
4	Björling	2019	Anxiety and depression	No	No	No	Yes	No	Yes	No	5
5	Brooks	2021	Mental health literacy	Yes	Yes	No	Yes	No	Yes	No	3
6	Cheng	2021	Mental healthcare	Yes	No	No	Yes	No	Yes	Yes	3
7	Christie	2019	Digital intervention	No	No	No	Yes	No	Yes	No	5
8	Culbong	2022	Mental health service provision	Yes	No	No	Yes	No	No	Yes	2
9	Davison	2022	Wellbeing (psychometric study)	Yes	No	No	Yes	No	No	Yes	2
10	Edridge	2018	Mental health service provision	Yes	Yes	No	No	No	No	No	1
11	Gabrielli	2020	General mental health	No	No	No	Yes	No	Yes	Yes	4
12	Gellatly	2019	Mental health of those with a parent with serious mental illness	Yes	No	No	Yes	No	Yes	No	4
13	Gobat	2021	Whole‐school approaches to mental health	Yes	No	Yes	Yes	Yes	Yes	Yes	5
14	Gonsalves	2019	Conduct problems, anxiety, depression	Yes	No	No	Yes	No	Yes	No	4
15	Grové	2021	Depression and anxiety	Yes	No	No	Yes	No	No	Yes	2
16	Hackett	2018	Mental health service provision	Yes	No	No	Yes	No	No	Yes	2
17	Hill	2022	Anxiety	Yes	No	Yes	Yes	Yes	Yes	No	6
18	Hugh‐Jones	2022	Mental health support in schools	Yes	No	Yes	Yes	Yes	Yes	No	6
19	Hugh‐Jones	2023	Virtual reality intervention	Yes	No	No	Yes	No	Yes	No	4
20	Latif	2017	Self‐harm	No	No	No	Yes	No	No	Yes	3
21	Li	2022	Depression and anxiety	Yes	No	Yes	Yes	Yes	Yes	No	6
22	Libon	2022	Suicide prevention	Yes	No	No	Yes	No	No	Yes	2
23	Mindel	2021	Digital mental health service	Yes	No	Yes	Yes	Yes	Yes	Yes	5
24	Moltrecht	2022	Emotion regulation and mental health	Yes	No	No	Yes	No	Yes	No	4
25	Morote	2022	General mental health	Yes	No	No	Yes	No	Yes	Yes	3
26	Neill	2022	General anxiety and test anxiety	Yes	No	No	Yes	No	Yes	No	4
27	O’Brien	2022	Eating disorders	Yes	No	No	Yes	No	Yes	No	4
28	Povey	2020	Digital intervention	Yes	Yes	No	Yes	No	Yes	No	3
29	Povey	2022	Mental health app development	Yes	Yes	Yes	Yes	Yes	Yes	No	5
30	Realpe	2020	Intervention (psychosis)	Yes	No	No	Yes	No	Yes	No	4
31	Syed Sheriff	2022	Online intervention development	No	No	No	Yes	No	Yes	Yes	4
32	Stoyanov	2021	Mental health support	Yes	No	Yes	Yes	Yes	Yes	Yes	5
33	Thomson	2022	General mental health	Yes	Yes	No	No	No	No	Yes	0
34	Thorn	2020	Suicide prevention	No	Yes	Yes	Yes	Yes	Yes	Yes	5
35	Warne	2022	Genes and mental health	Yes	No	No	Yes	No	No	No	3
36	Zieschank	2021	General mental health	No	No	No	Yes	No	Yes	Yes	4
Percentage of studies with favorable features				22%	74%	24%	96%	24%	70%	36%	

*Note*: green = unproblematic; yellow = potentially problematic; red = problematic.

Abbreviation: PPI, Patient and Public Involvement.

### Replicability

The most positive finding across all studies was the transparency of the various roles of PPI from design to dissemination, presented in some detail in 96% of the studies. Roles ranged from involvement in all stages of the research to involvement in a minor, advisory part during sample recruitment. More problematic from a reproducibility perspective were the profiles of the participants involved. Most studies lacked clarity about the recruitment, number, age, gender, and SocioEconomic Status/ethnicity. Only 24% of the studies provided necessary information about the PPI representatives. No PPI was based on a random selection of participants or patients from a well‐defined population. In several cases, the number of PPI representatives was rather small, less than 10 individuals in 22% of the studies (see Table 2). Because of lack of transparency in a critical part of the PPI (presenting their recruitment, age, gender, and background characteristics) potential replicability of the large majority of the studies (76%) was coded as problematic.

### Translation

In most cases the authors emphasized the study's exploratory aims and suggested further examination of the impact of PPI and the effectiveness of the interventions based on PPI research (70%). In several cases RCTs were mentioned as a necessary next step. For example, Hugh‐Jones et al. (2023) proposed that their prototype of a virtual reality intervention for youth mental health problems, developed with PPI, would be ready for pilot testing. They refrained from recommendations for policy or practice. Yet, not all authors limited their recommendations to further research. In their study developing an app to support youth with mental health problems, Stoyanov et al. (2021) proposed further examination of the quality and efficacy of the app in a series of RCTs. At the same time, however, they reported to have advised policymakers and to put the app (Niggle) on the World Wide Web.

In the majority of studies (64%) the translation to policy or practice was done in the absence of replicated or even replicable results. Bristow and Atkinson (2020)state on the one hand that “Repeating the design at another school might yield different results,” but on the other hand made the general recommendation to implement holistic playtime provision. In another study the authors argued that further research was needed for a more in‐depth evaluation of the efficacy of the intervention with help of PPI, while they also considered their treatment as scalable (Gabrielli et al., 2020). Morote et al. (2022) called for experimental and longitudinal designs to provide stronger evidence of causal claims, but also included translational recommendations. Developing an online arts and culture intervention for troubled youth, Syed Sherif et al. (2022) admit that “the design would have benefitted from a third arm to evaluate efficacy compared to a waitlist control” but they are confident that “there now is early evidence for the great potential of this online approach for supporting youth mental health.”

### Co‐researchers and co‐authors

A minority of the PPI study reports (22%) included PPI representatives in the list of co‐authors. As an example, the participatory action research by Ennals et al. (2022) aimed at a co‐production with residents and staff of a youth residential rehabilitation center improving the chances of a better treatment. The steering group including representatives of residents and staff was indeed involved in all stages of the year‐long project, leading to insights into how to facilitate a safe space that supports young patients to get involved in the change work needed “to heal and grow.” The findings were considered to offer guidance to create such an enabling milieu in other settings as well. This combination of having a role as co‐researcher and a co‐authorship was observed in 24% of the studies (see Table 2). A second example is the participatory study of Kendal et al. (2017) aimed at elucidating the needs for emotional support in young people, in particular through the Internet. The participants were included in all stages of the research and their contributions were acknowledged in a collective authorship, named “co‐researchers' group” with a footnote presenting their names.

The percentage of studies in which PPI representatives were co‐researchers but not co‐authors amounted to 54%. As an example, the 3‐year participatory action research by Culbong et al. (2022) on improving mental health service provision to young Aboriginal people in Western Australia involved Aboriginal elders, young people, and non‐Aboriginal mental health service and youth policy staff in co‐designing a culturally‐adequate model of care. Co‐designing a care framework did not result from just asking participants about their mental health needs and how they could be met, but was the outcome of close cooperation and communication between various stakeholders, including youth, involved as providers or receivers of care for mental health issues. Eight Aboriginal young people and seven Aboriginal elders were mentioned by name in the Acknowledgments as co‐designers of the study. The first two authors of the paper were from indigenous families and had an academic background.

In only one study (2%) PPI representatives with an academic background were co‐author but with limited involvement in data‐collection and coding (Thorn et al., 2020). In 10 studies (20%) PPI did not involve a role as co‐researcher and did not lead to a co‐authorship. For example, Zieschank et al. (2021) reports on developing an assessment device for self‐reported digital distress by 5‐ to 11‐year‐old Australian children. In interviews the children were asked to evaluate animations of questions on digital distress to make the assessment more feasible and understandable for this age group. The children served as source of information but did not act as co‐researcher in any methodological sense of this term. The authors of the paper were all academics, so this study seems more similar to a conventional study with strict role division between respondents and researchers than an example of participatory research using PPI.

## DISCUSSION

In this study, we examined the state of the art in PPI youth mental health research, in particular with an eye to transparency and replication as necessary conditions for translation. Based on our systematic coding of PPI studies, we found that almost two‐third of the 50 studies on youth mental health incorporating PPI (suggest to) translate their results to policy or practice, mostly as recommendations but sometimes also by dissemination of (online) interventions. At the same time the authors of a substantial majority of the studies (70%) also suggest the need for further work on their results, for example, proposing RCTs to validate the outcome of their exploratory inquiry. If we want responsible translation of scientific results to policy or practice, the target populations (patients, parents, teachers, medical professionals, therapists) need to be confident that the results are not just preliminary findings or hypotheses but robustly replicated findings. Translation requires replication, replication requires reproducibility, and reproducibility requires transparency. Only a quarter of the PPI studies was replicable. As a consequence, a large majority of the PPI studies suggest premature translation of their results. An example of such premature translation is Thorn et al.’s (2020) PPI‐based study on the development of an online suicide prevention program, rolled out across Australia through a social media campaign. The authors themselves consider the program as lacking clear evidence for effectiveness, as they conclude: “**If effective**, the campaign has the potential to better equip many young people worldwide to talk safely on the web about suicide.” (Thorn et al., 2020, bold added). The problem is that rolling out a preventive intervention without knowing its effectiveness may be a waste of time and resources and may create unforeseen collateral damage to vulnerable individuals, in particular in case of suicidal youth.

### Replication

At least two characteristics of PPI studies seem to hamper replication. The first problem is lack of transparency of the PPI component itself. We found that most studies are rather clear about the roles of PPI in the research cycle. However, the descriptions of the individuals constituting PPI often remained incomplete. A next PPI study on the same theme but including a different set of individuals would run the risk of getting different results, certainly when PPI is also assigned a decision‐making role in several stages of the research cycle. In the 50 PPI studies examined here we did not find even one that had implemented replicable, random recruitment of PPI individuals from a well‐defined population. We propose that PPI may follow the example of the “citizen assembly” (Van Reybrouck, 2016) based on sortition or stratified random selection that already has been successfully applied to seemingly intractable political issues (Van IJzendoorn & Bakermans‐Kranenburg, 2021). The second problem for replicability, related to the more radical versions of PPI such as participatory action research, is the merging of research and activism from start to finish of the project. Such investigations are inherently non‐replicable because at any point or stage in the process of data‐collection or analysis an activist decision might result in a radical turn in a different, strategically more promising scientific direction. Assigning PPI representatives the role of co‐researchers with decision‐making responsibilities creates similar risks with replicability.

At the extreme end of the PPI continuum the approach becomes entangled in a web of contradictions between ideal and real implementations. Reflections on the epistemological and sociological background of actual (community‐based) participatory action research projects led Janes (2016) to doubt the possibility of going beyond the power difference between community participants and researchers. Already talking about “giving voice” to youth or other participants would be emphasizing the superiority of the academic worker, a superiority which is exacerbated by the asymmetrical financial and social privileges of the academic versus community representatives (see also Fox, 2013). Janes' (2016) conclusion is that instead of the “**tyranny of everyone participating in everything**” a better model would be to stress the productive complementarity of different expertise and skills—which aligns well with our proposal of a Cooperative Practitioners‐Researchers Model (see Figure 1). On similar grounds, Fox (2013) drew a rather different conclusion from an exploratory participatory study that uncovered the dynamics of resistance of the target group against a participatory role. According to Fox (2013), the balance between academic researchers and participating youth should radically shift toward an exclusive decision‐making role of the participants, nearly to the exclusion of any researcher input. The risk is that these participants' voices would be considered to represent the larger community group although they might have been selected only because they were the most articulate and easily accessible individuals (Hayward et al., 2004).

### PPI in the research cycle

PPI might be a valuable asset in the research cycle if it is restricted to specific parts of this cycle. In our Cooperative Practitioners‐Researchers Model (Van IJzendoorn & Bakermans‐Kranenburg, 2024) we propose that practitioners (participants, patients, clinicians or policy makers) may be involved in the first and in the final stages of the research process (see Figure 1). They may be involved in initiating the research topic or help to formulate relevant research domains and questions, which the researchers translate into testable research questions and hypotheses, linking them to valid measures, adequate designs and data‐analytic plans. At the end of the research process, participants may advise researchers on how to interpret the findings, with a focus on the translational value and ethical implications of the results, and to support implementing the findings in a responsible way in policy or practice. The Cooperative Practitioners‐Researchers Model is based on Habermas' critical theory of the three knowledge interests (“Erkentnissinteressen,” Habermas, 1968; Bohman & Rehg, 2017) which play indispensable roles in modern science and its translation: a practical interest in finding correct interpretations of participants' perspectives and needs, a technical interest in finding effective means to specific goals, and an emancipatory interest in open communication about justifiable ways to connect means to desirable ends.

In this Cooperative Practitioners‐Researchers Model the roles and responsibilities of PPI representatives and researchers are clearly differentiated. Researchers should focus on testing and trying to refute grounded hypotheses about predictive or causal associations in the context of justification (Popper, 1959) in a transparent and replicable way. In contrast, PPI centers on proposing global research questions and suggesting bold conjectures in the context of discovery (Popper, 1959), and supports ethically responsible translation of replicated findings to policy or practice. With this division of roles PPI is not regulated by stringent methodological rules and conventions, in the context of discovery “anything goes” (Feyerabend, 1975). In the translational stage a strictly scientific jump from description or explanation (“is”) to what should be done (“ought”) is impossible. Normative or strategic input is needed (see for elaboration Van IJzendoorn & Bakermans‐Kranenburg, 2024). Within the boundaries of the law on (mal‐)treating youth or PPI participants anything goes in PPI in youth mental health research as long as PPI studies are considered to produce hypotheses to be tested in rigorous research and PPI representatives support translations only of replicated results to policy or practice.

Considering more radical forms of PPI such as participatory action research in the context of discovery also solves the conundrum of their self‐imposed lack of transparency, reproducibility and replicability. As Cornish et al. (2023) argue, the principle of reproducibility may not be functional in this type of collaborative, open‐ended and unpredictable process that is difficult to monitor and to describe in detail, let alone to emulate in any realistic or virtual way. Of course, for almost every empirical study the argument can be made that data‐collection takes place in a specific socio‐cultural and relational context and thus exact replication would be impossible. Reproducibility however is the less demanding theoretical possibility to trace the steps of the investigators based on their description of the process of inquiry (National Academies of Sciences, Engineering, and Medicine, 2019). But for participatory action researchers it would be impossible to participate in open science, sharing anonymized or pseudonymised data with other research teams or the wider public because “marginalised” activists or communities would not be exposed to “exploitation by university researchers” (Cornish et al., 2023). Such an isolated “closed science” is difficult to defend, except if what matters only is the outcome of the inquiry in terms of a set of hypotheses for scrutiny in the necessarily open context of justification.

### Translation

Elsewhere we argued that for responsible translation two other requirements are necessary besides replicated findings, namely a viable cost‐benefit or cost‐utility balance and justifiable ethical arguments to bridge the gap between “is” and “ought” (Van IJzendoorn & Bakermans‐Kranenburg, 2024). In several PPI papers the extra expenses related to the involvement of youth were mentioned but an honorarium was usually not recompensated via the grants. In their paper on the Youth PPI Cafe, Thomson et al. (2022) therefore complain about the low funding priority of PPI, despite its potential cost‐effectiveness. One of the few cost‐utility evaluations of PPI in youth mental health research, however, showed a somewhat disappointing balance (Hugh‐Jones et al., 2022).

The other requirement is the presence of an ethical bridge between replication and translation. In several PPI papers ethical issues are addressed but in most cases they pertain to the way in which the PPI representatives are being treated. Remarkably, Research Ethics Boards not always require consent of PPI representatives because they would not be involved in research (Moltrecht et al., 2022). Yet, what we also mean here is convergence between researchers and target group on the desirability of the aims of an intervention. In the Abel et al. (2020) feasibility RCT to improve quality of life in children of parents with serious mental illness, the authors provided evidence that their Young Simplifying Mental Illness plus Life Enhancement Skills intervention was concordant with the parents' ethical views, an important first step to ethical justification of intervention translation to (clinical) practice. From a translation perspective we have argued that PPI shaped after a citizen assembly might built a firm foundation for the bridge between “is” and “ought” (Van IJzendoorn & Bakermans‐Kranenburg, 2024).

### Authorship

Concrete guidelines for (co‐)authorship or other means of re‐compensation of the PPI representatives' investments would constitute an important component of transparent and justifiable treatment of participants. This is a delicate issue because it touches on the concept of “author” in scientific research. Etymologically the term is derived from the Latin “auctor” which alludes to originator, initiator or designer (“auctor intellectualis”) but in our era of big science with large teams of hundreds of scientists a (co‐)author might not even have actually written any part of the article, except maybe their own name. Full accountability for all ingredients of an interdisciplinary study with complicated measurements and analyses might be too much to ask from almost every scholar. Nevertheless, every (co‐)author should be accountable for the correct application of more general guidelines for sound research such as starting with testable and refutable hypotheses, using valid assessments of central constructs, collecting and coding data in a transparent way, conducting reproducible analyses, and reporting findings without overstating the implications of the collected data. The scientific mindset or habitus required for such a critical overview of the research process is part of an academic education and of “lived experience” in research projects. In fact, ideal PPI representatives might be individuals with both lived experiences in research and in the substantive domain of inquiry (see for an example Culbong et al., 2022). Most PPI representatives do not fulfill these criteria and thus asigning co‐authorship would probably be at odds with current American Psychological Association or International Committee of Medical Journal Editors guidelines for authorship. “Anything goes” does not apply to co‐authorship of participants or patients who are involved in a PPI study.

The question of PPI authorship touches on a more fundamental problem of equality between participants and researchers. Striving for equality in studies with a PPI ambition is sometimes interpreted as leveling the differences between the scientific knowledge of researchers and experiential knowledge of the participants. Without level playing fields for both parties the structural power differential in favor of the academic researchers would be continued at the expense of the (youth) participants. The implication would be that academics should give up their privileged positions and asymmetrical financial compensation (Janes, 2016) and be satisfied with far less recognition for academia (Fox, 2013). Publications on participatory research should not be authored solely by academics (but see Cornish et al., 2023) and “giving voice” instead of structural autonomy to participants should indeed be considered a haughty “postcolonial” stance (Janes, 2016). The aim of a power redistribution may only marginally be served by using “role swapping to distribute the leadership roles of chairing meetings” (Cornish et al., 2023). Even training of participants in research skills, one of the “nonnegotiables” of youth participatory research (Ozer, 2016) runs the risk of implicitly prioritizing and at the same time undervaluing (Janes, 2016) academic knowledge and expertise.

But equality of roles in studies with PPI might also be interpreted in the sense of complementary and equivalence knowledge and skills assigned to different stages in the research—translation cycle. In our Cooperative Practitioners‐Researchers Model, the input of researchers in the stages of designing and conducting a study in a transparent and reproducible way is indispensable, as is the input of participants' experiential knowledge in the stages of developing a research question and in the translational interpretation of research results. The epistemic position of participants might be strengthened by a more careful selection procedure of a larger and more representative number of participants to avoid relying on only a few vocal individuals who are most readily available (Fox, 2013; Hayward et al., 2004). A “citizen assembly” approach with stratified selection to include a diversity of participants might work for larger research programs. In smaller projects the American jury model may be used for creating an unbiased “PPI pool” or “venire” from which a diverse sample of participants can be selected with the best fit for the specific study demands. A substantial stipend per day invested by participants would be required and should be made available by funding agencies (Cornish et al., 2023). The provision of psychological support for “lived experience” participants in studies on family violence, maltreatment or other potentially traumatizing experiences is an evident ethical requirement (Ozer et al., 2020).

## CONCLUSIONS

PPI representatives might have important advisory roles in the research cycle, in particular in the exploratory stage of selecting a research question and piloting the development and feasibility of designs to be tested in further replicable studies. PPI might also support the translation of replicated findings to policy or practice. Anything goes for PPI in exploratory research. Whether PPI really adds to the scientific validity and impact of a project as Racine et al. (2023) and many others argue, remains to be seen (Ozer et al., 2020) or rather, to be tested through randomized trials or related methods. The outcome of such scrutiny might not always confirm widely shared assumptions, as Crocker et al. (2018) showed in their meta‐analysis of the impact of PPI in clinical trials on recruitment (small positive impact) and retention (no influence).

More evidence from randomized trials on translational impact of PPI in studies of youth mental health is badly needed before grant foundations such as Wellcome Trust, United Kingdom Research and Innovation or National Institute for Health and Care Research in the UK should expect or even require PPI in promising or fundable grant proposals. Forcing researchers to use PPI to reach ambitious translation goals might run the risk of hasty translation, collateral iatrogenic damage (an ethical issue) or local non‐sustainable impact (negative cost‐benefit balance), without substantial contribution to scientific evidence. Radical merging of exploratory research and premature action in participatory action research seems incompatible with replicable and therefore translatable inquiry. For translation of the findings of PPI studies to policy or practice a firm evidence base of replicated results is needed, which can only be delivered by slow science with investments for the long haul.

## AUTHOR CONTRIBUTIONS

**Marian J. Bakermans‐Kranenburg**: Conceptualization; data curation; formal analysis; investigation; methodology; validation; visualization; writing—original draft; writing—review and editing. **Marinus H. van IJzendoorn**: Conceptualization; data curation; formal analysis; investigation; methodology; project administration; resources; validation; visualization; writing—original draft; writing—review and editing.

## CONFLICT OF INTEREST STATEMENT

The authors have declared that they have no competing or potential conflicts of interest.

## ETHICAL CONSIDERATIONS

No ethics approval needed, no participants or patients involved. No permission required to reproduce material from other sources.

## Data Availability

Primary data already in the public domain.
